# Assessing Autophagy in Sciatic Nerves of a Rat Model that Develops Inflammatory Autoimmune Peripheral Neuropathies

**DOI:** 10.3390/cells6030030

**Published:** 2017-09-18

**Authors:** Susana Brun, Nicolas Schall, Hélène Jeltsch-David, Jérôme de Sèze, Sylviane Muller

**Affiliations:** 1 INSERM UMR_S 1119, Biopathologie de la Myéline, Neuroprotection et Stratégies Thérapeutiques, Faculté de Médecine/University of Strasbourg, 67000 Strasbourg, France; susana.brun@unistra.fr (S.B.); jerome.de.seze@chru-strasbourg.fr (J.S.); 2 CNRS UPR3572, Immunopathologie et Chimie Thérapeutique/Laboratory of excellence Medalis, Institut de Biologie Moléculaire et Cellulaire, 67000 Strasbourg, France; n.schall@ibmc-cnrs.unistra.fr (N.S.); hdavid@unistra.fr (H.J.-D.); 3 Department of Neurology, Hôpital de Hautepierre, 67000 Strasbourg, France; 4 Institute for Advanced Study, University of Strasbourg (USIAS), 67000 Strasbourg, France

**Keywords:** macroautophagy, chaperone-mediated autophagy, peripheral neuropathies, murine models, rat sciatic nerve, CIDP

## Abstract

The rat sciatic nerve has attracted widespread attention as an excellent model system for studying autophagy alterations in peripheral neuropathies. In our laboratory, we have developed an original rat model, which we used currently in routine novel drug screening and to evaluate treatment strategies for chronic inflammatory demyelinating polyneuropathy (CIDP) and other closely related diseases. Lewis rats injected with the S-palmitoylated P0(180-199) peptide develop a chronic, sometimes relapsing-remitting type of disease. Our model fulfills electrophysiological criteria of demyelination with axonal degeneration, confirmed by immunohistopathology and several typical features of CIDP. We have set up a series of techniques that led us to examine the failures of autophagy pathways in the sciatic nerve of these model rats and to follow the possible improvement of these defects after treatment. Based on these newly introduced methods, a novel area of investigation is now open and will allow us to more thoroughly examine important features of certain autophagy pathways occurring in sciatic nerves.

## 1. Introduction

Autophagy is a vital biological lysosome-mediated process that maintains cellular homeostasis by degrading abnormal proteins and organelles. Three major types of autophagy have been recognized, including macroautophagy, chaperone-mediated autophagy (CMA), and microautophagy [1,2]. Macroautophagy (often just called autophagy) is the most common form of autophagy, and during this process, the cell forms a double-membrane sequestering compartment termed the phagophore, which matures into the autophagosome. Following delivery to the vacuole or lysosome, the cargo is degraded and the resulting macromolecules are released back into the cytosol for reuse [3,4]. CMA is the most selective type of autophagy, and involves the translocation of cytosolic proteins containing a specific degradation signal (the KFERQ sequence motif) to the lysosomal lumen. This motif, found in around 30% of cytosolic proteins, is usually buried in the fully folded protein but can be exposed upon partial unfolding. It is recognized by HSPA8/HSC70 chaperone which, together with other co-chaperones, targets the protein to the CMA adaptor (LAMP2A) localized on the lysosomal membrane. This interaction induces unfolding and translocation of the targeted protein into the acidic lysosomal lumen, followed by their degradation by lysosomal enzymes, a large family of enzymes that encompass some glycosidases, proteases (e.g., cathepsins), sulfatases, and others [5,6,7]. Microautophagy is by far the least understood phenomenon. It is characterized by the direct invagination of the lysosomal membrane to sequester cytoplasmic cargo [8,9]. This non-selective lysosomal degradative process maintains organelle size, membrane homeostasis, and cell survival under nitrogen restriction. Noticeably, these three main autophagy pathways—macroautophagy, CMA, and microautophagy—are remarkably interconnected and further coordinated with other self-eating pathways.

A number of recent studies tend to establish a link between possibly altered autophagy processes and several autoimmune [10,11,12,13,14,15,16,17], and especially autoimmune, neurological diseases [18,19,20,21] (Table 1).

However, it has not been well addressed whether autophagy is affected in inflammatory/autoimmune peripheral neuropathies such as Guillain–Barré syndrome (GBS) and chronic inflammatory demyelinating polyneuropathy (CIDP). GBS and CIDP are, respectively, the human acute and chronic inflammatory demyelinating disorders of the peripheral nervous system (PNS), which are clinically characterized by an involvement of proximal as well as distal limb structures (weakness, paraesthesia). In these pathologies, the disease is presumably caused by damage to the myelin sheath (i.e., a wrap of myelin that surrounds axons) of the peripheral nerves [20,21]. The most widely used animal model of GBS is the experimental autoimmune neuritis (EAN) and recently, we developed and characterized a new representative rat model for human CIDP, the chronic-EAN [26,27]. Because the pathophysiology of GBS, and particularly of CIDP, remains poorly understood, these models represent important tools to progress in the knowledge of the pathogenesis of these diseases and for translational drug studies.

## 2. Rat Sciatic Nerve as a Valuable Model to Study Autophagy

The rat sciatic nerve is commonly studied by researchers working on peripheral neuropathies not only for its anatomical features, but also for it length and consequently, the amount of tissue available for immunohistochemical, western blotting, and PCR experiments, for example. Furthermore, it seems that it represents one of the first nerves to be committed in EAN and in chronic-EAN, adding value to this model of choice. 

**Figure 1:**
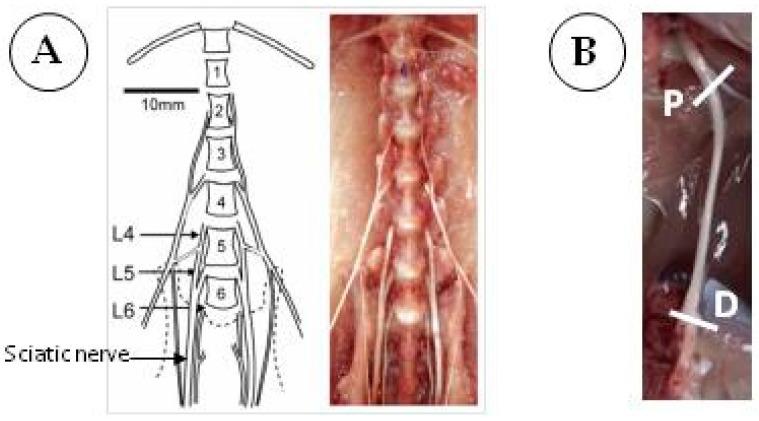
Dissection of rat sciatic nerves. (**A**) Ventral view of dissection and reference drawing made from it, showing the segmental origins of sciatic nerve from Sprague Dawley strain (figure taken from Rigaud et al. [30] with permission); (**B**) Sciatic nerve from a male Lewis rat aged nine weeks showing removal sites of the proximal (P) and distal (D) segments.

In rats, the sciatic nerve originates from the fusion of spinal segments L4-L6 but principally from L4 and L5 spinal nerves [28,29,30] to form the nerve on the lesser pelvis (Figure 1A). Only one study demonstrated that the components of sciatic nerve from Sprague Dawley rats vary from L3 to L6 [31]. After the sciatic nerve has left the pelvis, it curves around the greater trochanter as a single fascicle (considered as the proximal segment of the sciatic nerve), then takes a descending path to splits into two and then into four fascicles. The segment immediately before the terminal branching is considered the distal segment (Figure 1B).

Taking into consideration all these characteristics, the rat sciatic nerve undoubtedly represents an adequate model for studying fundamental questions as the one we undertook to examine autophagy in peripheral neuropathies. This area has been seldom investigated in sciatic nerves and therefore there is very few examples of methodological precedent to rely on. However, there are many papers describing appropriate techniques to assess autophagy and notably to measure the autophagy flux in organs and diverse tissues [18,32,33,34,35]. Therefore, we took advantages of these studies and adapted existing protocols to our specific material. Our work was also based on immunofluorescence and western immunoblotting methods to test in rats the ex vivo alterations of molecules implicated in autophagy [19,36,37,38,39]. Transmission electron microscopy (TEM) allows the monitoring of autophagic vacuoles and autophagosomes [37,38,39].

As indicated above, there are nowadays no investigations on autophagy processes in GBS and CIDP. The only related study on inflammatory demyelinating polyneuropathies was made by immunoblotting in EAN, the animal model mimicking the classical monophasic acute form of GBS [19]. In this recent study, expression levels of autophagy markers Beclin-1, a component of the phosphatidylinositol-3-kinase complex that mediates vesicle-trafficking processes, and of microtubule-associated protein 1 light chain 3 (MAP1LC3B), a member of the highly conserved ATG8 protein family were significantly increased. The MAP1LC3B-II/I ratio was also found to be increased. Concomitantly, as expected from these data, the expression level of sequestosome-1 (SQSTM1), also known as the ubiquitin-binding protein p62, was decreased in sciatic nerves of EAN rats compared with control rats. In full agreement, when examined by TEM, the formation of autophagosomes in axons and myelin sheaths of sciatic nerves in EAN rats was increased, compared with the control group. Together, these data suggest that autophagy activity is increased in the sciatic nerve of EAN rats.

The rat sciatic nerve has also been used to study autophagy in other pathologies of the PNS such as peripheral nerve injury [39] and peripheral neuropathy occurring in patients with metabolic syndrome [37] and diabetes [38]. In these studies, it should be underlined that alterations evaluated in western blot of MAP1LC3B-I and -II, Beclin-1, ATG5 and ATG7 expression, and MAP1LC3B-II/I ratio were demonstrated, whereas some variations of MAP1LC3B-I and -II expression only were detected by immunofluorescence. TEM was used for detecting the presence of autophagosomes. These observations further support the view that multiple methodological approaches have to be combined to ascertain the eventual perturbations of autophagy in any tissues or naive biological materials [34].

The aim of this short technical review is to summarize the contribution of different experimental techniques used to study autophagy in ex vivo sciatic nerves from rats developing an immune peripheral neuropathy.

### 2.1. Induction of CIDP in Rats

**Figure 2:**
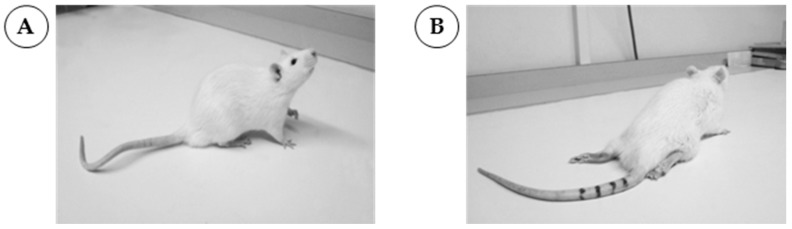
Visualization of clinical scores from male Lewis rat aged nine weeks. (**A**) Rat immunized with CFA and not developing clinical sign (clinical score of 0); (**B**) Rat immunized with S-palm-P0(180-199) peptide plus CFA and developing CIDP (clinical score of 3). Abbreviations: CFA, complete Freund’s adjuvant; CIDP, chronic inflammatory demyelinating polyneuropathy.

For inducing the disease, we use male Lewis rats, 7–8 weeks old, weighting 250–270 g. They receive subcutaneously, at the base of the tail, 200 μL of an inoculum containing 200 μg of S-palm-P0(180-199) peptide (sequence: Acetyl(palm)KRGRQTPVLYAMLDHSRS) and 0.5 mg of *M. tuberculosis* (strain H37 RA, Difco, Detroit, MI, USA) emulsified in 100 μL of saline and 100 μL of incomplete Freund’s adjuvant (Sigma-Aldrich, St. Louis, MO, USA) [26,27]. Rats that received complete Freund’s adjuvant (CFA) alone are used as control. Body weight and clinical scores are assessed daily from day 0 until day 60 days post-immunization. Severity of paresis is graded as follows: 0—no illness; 1—flaccid tail; 2—moderate paraparesis; 3—severe paraparesis; 4—tetraparesis; 5—death (Figure 2).

### 2.2. Immunofluorescence

Immunofluorescence is a common laboratory technique, which is based on the localization of specific antigens using antibodies conjugated to fluorescent dyes (fluorochromes). 

CIDP is pathologically characterized by focal inflammatory demyelination followed by axonal degeneration. The prominent lymphoid cells in the lesion are macrophages, which actively contribute to the demyelinating process. Our rat model mimicking CIDP fulfils these features. It was exploited to investigate if an alteration of autophagy markers is present in macrophages, neurofilaments, or Schwann cells of Lewis rat sciatic nerves by co-localization studies of these markers. 

#### 2.2.1. Procedure

Rats are deeply anesthetized with Ketamine/Rompun and sciatic nerves are dissected out, fixed in Bouin-Hollande solution and embedded in paraffin. Staining is performed on 5-μm cross-sections. After dewaxing, nonspecific binding sites are blocked with 5% (*v*/*v*) horse serum (Vector Laboratories, Burlingame, CA, USA) in phosphate-buffered saline (PBS) pH 7.4 for 30 min at room temperature (RT). Sections are then incubated for 1 h at RT with primary antibodies against MAP1LC3B (5 μg/mL; MBL, Nagoya, Japan), LAMP2A (5 μg/mL; Abcam, Cambridge, UK), and SQSTM1 (5 μg/mL; Abcam) for visualizing autophagy in double immunostaining with neurofilament 200 (NF200; 25 μg/mL; Sigma-Aldrich), ED1 (1.25 μg/mL; Serotec, Oxford, UK), and S100 (a generous gift we received from Said Ghandour, Strasbourg, France) for Schwann cells (Table 2). Antibody binding to tissue sections is visualized with Alexa Fluor 488 or Cy3-conjugated secondary antibody (1:1200; Jackson ImmunoResearch, West Grove, PA, USA) for 1 h at RT. Coverslips are mounted in AntiFade Poly-Mount (4′,6-diamidino-2-phenylindole) medium (Polysciences Inc., Warrington, PA, USA) for MAP1LC3B + NF200 labeling, and in AntiFade Poly-Mount medium with 4′,6-diamidino-2-phenylindole (DAPI; Polysciences Inc.) for the other labeling.

**Figure 3:**
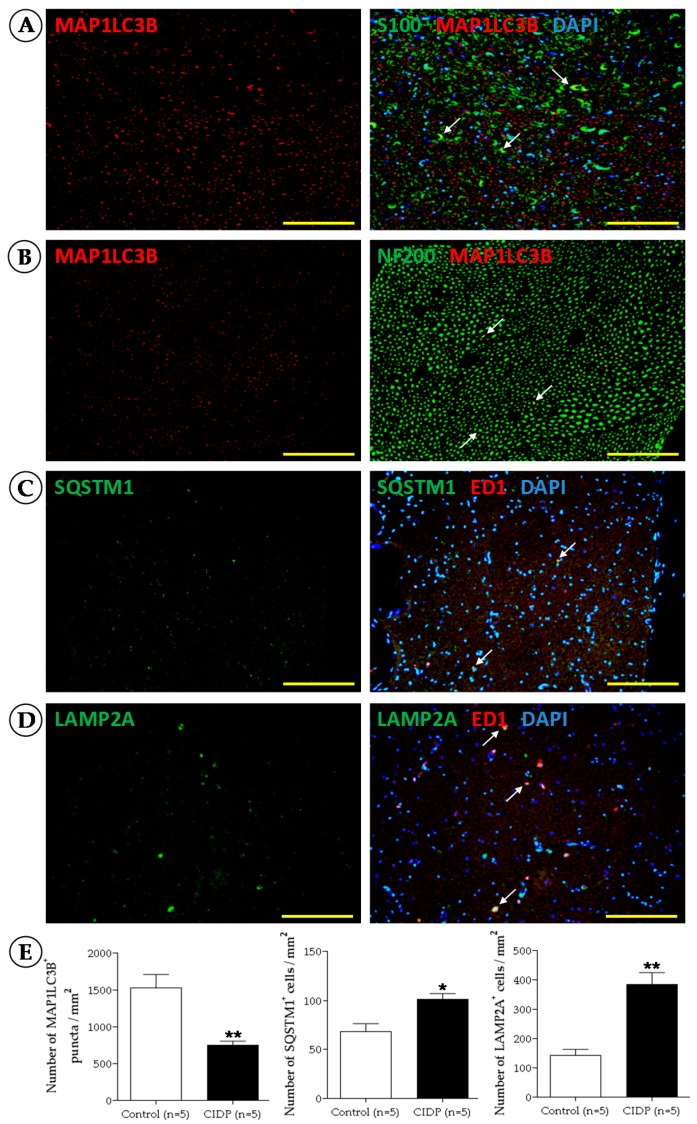
Representative immunofluorescence micrographs from transverse sections of sciatic nerve from an adult Lewis rat. (**A**) Staining of MAP1LC3B (red) and double staining of MAP1LC3B (red) with S100 positive Schwann cells (green); (**B**) Staining of MAP1LC3B (red) and double staining of MAP1LC3B (red) with neurofilament 200 positive nerve fibers (green); (**C**) Staining of SQSTM1 (green) and double staining of SQSTM1 (green) with macrophages (red); (**D**) Staining of LAMP2A (green) and double staining of LAMP2A (green) with macrophages (red). Nuclei are counterstained with DAPI (blue). White arrows show double staining. Yellow bar = 50 μm; (**E**) Analyses of staining are represented by histograms. Mean values and SEM are indicated; * *p* < 0.05, ** *p* < 0.01 compared to control.

Immunofluorescence staining is monitored with an Olympus BX60 fluorescent microscope and images are captured with the digital camera DP7V. Other devices are equally effective. To obtain quantitative analysis of the staining, in our experiments, the number of neurofilaments; macrophages; Schwann cells; MAP1LC3B, LAMP2A, and SQSTM1 positive cells; and the number of double-labeled spots are counted from a randomly selected 160 × 160 μm-area for MAP1LC3B labeling or 340 × 340 μm-area for the other labeling from five sections using the ImageJ software (Wayne Rasband, National Institutes of Health, Rockville, MD, USA). Representative images of the number of stained cells per mm^2^ are shown in Figure 3.

#### 2.2.2. Notes

Fixation with Bouin-Hollande solution is better than PFA because morphology and epitopes are well preserved. 

To reduce possible background, it is advisable:

(1) to use normal horse serum or normal donkey serum as blocking agent in PBS-Tween (PBS-T) rather than bovine serum albumin or fetal bovine serum;
(2) to use the primary antibodies in blocking solution;
(3) to use the secondary antibody in PBS-T and not in blocking solution;
(4) to wash in PBS and not in PBS-T.

It is crucial to fix the excised sciatic nerves very rapidly. As is well-known, fixation is critical for preventing autolysis and preserving cells and tissue components. This fact precludes the possibility of post-sawing incubation of neuronal tissues with lysosomal inhibitors for 4 h, which is required to inhibit lysosomal enzymes and therefore, the autophagic flux cannot be measured in this setting. The global autophagy markers expression levels only can be evaluated. 

#### 2.2.3. Advantages

One advantage of this method is that it allows the concomitant detection of two or three different antigens in a cell or the identification of a specific cell in a tissue by two or three antibodies labeled with different fluorophores, for example.

#### 2.2.4. Limitations

Some drawbacks have to be mentioned. In particular, auto-fluorescence remains a technical problem, which can produce false-positive results. Fluorochrome combinations must be carefully considered to limit emission spectra overlaps, especially for the detection of co-localized proteins. The fluorescence is not permanent and the photo bleaching phenomenon occurs as the samples are exposed to the light of microscope.

### 2.3. Western Blotting

Western blotting is a technique used to separate, identify, and quantify the amount of proteins present in cells or tissues, based on their respective molar mass by electrophoresis. Separated proteins are transferred onto a membrane that is then incubated with antibodies specific to the protein of interest.

#### 2.3.1. Procedure

**Figure 4:**
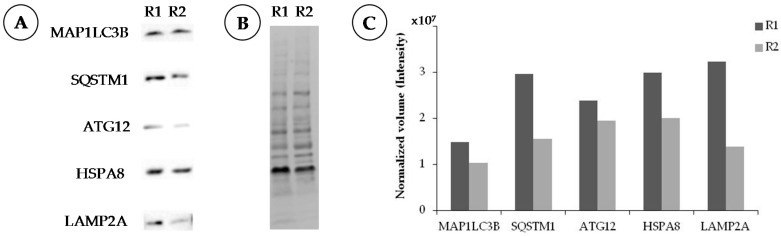
Western immunoblot analysis of sciatic nerve extracts from two adult Lewis rats R1 and R2. (**A**) Expression of autophagy markers MAP1LC3B, SQSTM1, ATG12, HSPA8, and LAMP2A; (**B**) Total protein used as normalization control visualized by stain-free technique; (**C**) Quantification of protein expressed as normalized volume (intensity).

Sciatic nerves are harvested, weighed, and homogenized with a tissue lyser in the so-called radio-immunoprecipitation assay (RIPA) buffer (150 mM NaCl, 50 mM Tris, 1 mM ethylene diamine tetraacetic acid (EDTA), 1% (*v*/*v*) Triton X-100, 0.5% (*v*/*v*) sodium deoxycholate, 0.1% (*v*/*v*) sodium dodecyl sulfate (SDS), pH 8) supplemented with protease inhibitors (Roche, Mannheim, Germany). Hundred μL of complete lysis buffer is added to 10 μg of tissue. Tissue homogenates are centrifuged at 12,000 g for 10 min at 4 °C and the supernatant collected to measure the protein concentration using the bi-cinchoninic acid assay Protein Assay Kit (Pierce Biotechnology, Rockford, IL, USA). Laemmli buffer (pH 6.8; BioRad, Hercules, CA, USA) containing 5% (*v*/*v*) β-mercaptoethanol) is added. Finally, lysates are denatured at 90 °C for 5 min and proteins (50 μg) are separated on a 4–20% Tris-glycine SDS-polyacrylamide gel electrophoresis gradient gel (Mini-PROTEAN^®^TGX Stain-Free™ protein gels, BioRad, Hercules, CA, USA) and then transferred onto nitrocellulose membrane (Trans-Blot^®^Turbo™ Transfer System, BioRad). Membranes are blocked with 3% (*w*/*v*) fat-free dry milk in Tris-buffered saline (TBS; pH 9.6) containing 0.1% (*v*/*v*) Tween-20 (TBS-T) at RT for 1 h. After one wash with TBS-T, antibodies specific to MAP1LC3B (0.5 μg/mL; MBL), SQSTM1 (0.5 μg/mL; Abcam), ATG12-ATG5 (1 μg/mL; Abcam), HSPA8 (0.5 μg/mL; Abcam), and LAMP2A (1 μg/mL; Abcam) are incubated overnight at 4 °C in TBS-T containing 1% (*w*/*v*) fat-free dry milk. After three washes with TBS-T, membranes are incubated for 1 h at RT with horseradish peroxidase-conjugated goat anti-mouse IgG fragment crystallizable (Fc) region-specific (50 ng/mL) and goat anti-rabbit IgG Fc fragment specific secondary antibodies (25 ng/mL; both from Jackson ImmunoResearch). After three further washes, a signal was detected using enhanced chemiluminescence reagent (BioRad) and Biorad-Chemiluminescence Imaging system. Protein levels are normalized by densitometry to total protein using the Image Lab^TM^ Software (BioRad). Typical results are illustrated in Figure 4. 

#### 2.3.2. Notes

Once sciatic nerves are dissected out, they must be immediately frozen at least at −80 °C until used. As is well known, defrosted samples are very sensitive to proteolytic degradation. As a result, it is not possible to introduce lysosomal inhibitors that should be added in half of the samples for 4 h to evaluate the autophagic flux and the total MAP1LC3B or SQSTM1 expression levels only can be measured at each given time. RIPA lysis buffer is better than lysis buffer (50 mM (4-(2-hydroxyethyl)-1-piperazineethanesulfonic acid (HEPES), 5 mM EDTA, 50 mM NaCl, containing 1% (*v*/*v*) Triton X-100, pH 7.4) because the amount of protein is more important. In our case, for the extraction of proteins from sciatic nerves, the utilization of a tissue lyser is better than using a Polytron or a potter homogenizer or than grounded to a power with a mortar and pestle in liquid nitrogen. In fact, with these last techniques, there is a significant loss of material and thus of protein.

Electrophoresis at 90 V for ~15 min and then at 120 V for ~1.5 min is essential to obtain correct bands signal when 50 μg of protein are charged. If a more important voltage is used, a band with a wave effect will be obtained. 

Using TBS-T for blocking and dilution solution is better than PBS-T because background is reduced. 

#### 2.3.3. Advantages and Limitations

Advantages of western blotting include:

- sensitivity (detection of small protein amounts);
- quantification of the amount of protein.

The limitations of western blotting are:

- high cost, time, and technical demand;
- requirement of numerous experimental optimizations (protein extraction, lysis buffer, blocking buffer, etc.);
- unexpected results: no bands, weak signal, unexpected or unusual bands, high background, etc.

### 2.4. TEM

TEM is an analytical method allowing visualization and analysis of specimens in the realms of microspace. It utilizes energetic electrons to provide morphologic, compositional, and crystallographic information. This technique has not yet been used in our laboratory to visualize autophagosomes but has been used by other researchers/research teams [22,34,37,38,39].

#### 2.4.1. Procedure

**Figure 5:**
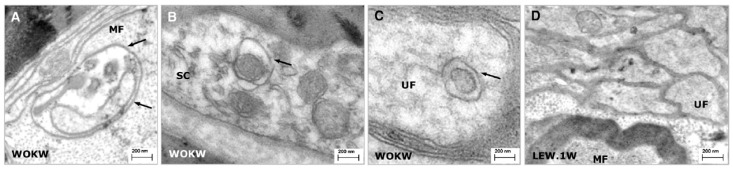
Ultrastructural images of sciatic nerves of five-month-old LEW.1W and WOKW rats. (**A**–**C**) Numerous autophagosomes (arrows) were found within the myelinated and unmyelinated nerve fibers and in Schwann cells of WOKW rats sciatic nerves; (**D**) Myelinated and unmyelinated nerve fibers visualized in sciatic nerves of LEW.1W rats (figures taken from Kosacka et al. [37] with permission). Abbreviations: LEW.1W, Lewis 1W; MF, myelinated fiber; SC, Schwann cells; UF, unmyelinated fibers; WOKW, Wistar Ottawa Karlsburg W.

Sciatic nerves are quickly removed and fixed with glutaraldehyde at a concentration from 2 to 3% (*v*/*v*) in the presence or not of 1% (*v*/*v*) of paraformaldehyde in 0.1 M PBS at 4 °C between 2 h and overnight. Then, the samples are post-fixed in 1% (*w*/*v*) osmium tetroxide in PBS for 1 h–1.5 h at 4 °C rinsed in PBS, dehydrated in a graded series of alcohol and propylene oxide, and embedded in Epon epoxy resin and polymerized at 60 °C for 48 h. Ultrathin sections (60–80 nm) are mounted on copper grids, contrasted with uranyl acetate and lead citrate, and visualized using a transmission electron microscope [22,34,37,38,39] (Figure 5).

#### 2.4.2. Note

According to our experience for other types of analysis, we recommend cutting transverse sections of sciatic nerves after glutaraldehyde fixation, especially if an analysis of myelin and axons is planned. Immunoelectron microscopy can be used to precisely evaluate the quantity and localization of autophagic markers MAP1LC3B and SQSTM1 at specific time points. 

#### 2.4.3. Advantages and Limitations

TEM is advantageous due to its powerful magnification, which can visualize intracellular details. However, there are also limitations to this approach: preparation of tissues or cells is very elaborate and lengthy, and the technique is very expensive. During the sample preparation process, human errors may lead to artifacts (e.g., the membrane continuity may be altered, organelles may be distorted or disorganized, etc.) that the experimenter must be able to differentiate from the true specimen structure. 

## 3. Conclusions

A number of comprehensive reviews and training materials have been published recently in the field covered in the present article. They specially highlight the fact that several independent methods have to be combined to adequately evaluate the autophagy activity status of considered cells and eventually convince ourselves of possible failures in these processes. We also highly recommend using a panel of these approaches as each presents specific bias, but when combined, they lead to data that reinforce each other to bring safer interpretation.

The methods described in this short technical review remain relatively traditional [18,34]. However, they are totally novel with regard to the tissues that are analyzed. Autophagy research in sciatic nerves is still in its beginning stages and further investigations will be crucial to precise the nature of defects occurring in diseases in which nerve cells of sciatic nerves are involved. Our hope is also that these investigations lead to the discovery of new autophagy markers, which may contribute detecting still unknown defects in some signaling circuits that might affect specific nerve cell subsets. The application could also be particularly valuable to follow the success of novel therapies based on molecules, peptides, biologics, or cells designed to target autophagy defaults in certain pathological settings. In this frame, following autophagy markers in a validated, robust, and sensitive program of relevant assays could be pertinent for managing stem cells—based treatments for sciatic nerve injury, for example. Associated to other already established criteria, these new assays might therefore contribute to a more accurate diagnosis of patients with peripheral neurological disorders, to a more selective recruitment of patients enrolled into clinical trials and for making decisions regarding their personalized treatment. It is worth remembering however that although pertinent animal models show similarities with human disease, it is likely that immunological responses are different because the complexity of human physiological immune system or because antigens involved in the pathology are not the same between the two species. Therefore, extrapolation to human disease should always be undertaken with caution and complementary studies need to be conducted.

## Figures and Tables

**Table 1:** Examples of autoimmune neurological diseases with autophagy abnormalities, and methods and model systems used for investigating these conditions.

Neurological Autoimmune Diseases	Autophagy Abnormalities	Methods	Model Systems or Patient Samples Tested	Ref.
Guillain–Barré syndrome (GBS)	Increased Beclin-1 and MAP1LC3B expression levels;	WB; TEM	EAN rat (sciatic nerves)	[22]
	Increased MAP1LC3BII/I ratio; Decreased expression of SQSTM1;			
	Increased formation of autophagosomes			
Chronic inflammatory demyelinating polyneuropathy (CIDP)	Increased Beclin-1 and MAP1LC3B-II expression levels;	TEM; WB	EAN rat (sciatic nerves)	[19]
	Increased ratio MAP1LC3B-II/I;			
	Decreased expression of SQSTM1			
Multiple sclerosis (MS)	Increased mRNA and protein level of ATG5;	qPCR; WB	EAE mice (blood) and patient (blood and brain)	[23]
	Decreased expression of *ATG16L2* and *ATG9A* genes;	qPCR	Patient (blood)	[24]
	Increased expression of *ULK1* gene			
Neuromyelitis optica (NMO)	Increased ATG5 variants	Mass array system	Patient (blood)	[25]

Abbreviations: ATG, autophagy related-gene; EAE, experimental autoimmune encephalomyelitis; EAN, experimental autoimmune neuritis; MAP1LC3B, microtubule-associated protein light chain 3; PCR, polymerase chain reaction; qPCR, quantitative PCR; RNA, ribonucleic acid; SQSTM1, sequestosome-1; TEM, transmission electron microscopy; ULK1, Unc-51 like-autophagy activating kinase 1; WB, western blotting.

**Table 2:** References of antibodies used in our settings to analyze autophagy activity by IF and WB in sciatic nerves from Lewis rats.

Antibody	Supplier; References	Technique (Concentration)
MAP1LC3B	MBL; M186-3	IF (5 μg/mL)
		WB (0.5 μg/mL)
SQSTM1	Abcam; ab109012	IF (5 μg/mL)
		WB (0.5 μg/mL)
HSPA8	Abcam; ab21052	WB (0.5 μg/mL)
ATG12	Abcam; ab155589	WB (0.5 μg/mL)
LAMP2A	Abcam; ab125068	IF (5 μg/mL)
		WB (0.5 μg/mL)

Abbreviations: ATG, autophagy related-gene; HSPA8, heat shock 70 kDa protein 8; IF, immunofluorescence; LAMP2A, lysosome-associated membrane protein 2; MAP1LC3B, microtubule-associated protein light chain 3; SQSTM1, sequestosome-1; WB, western blotting. Suppliers: Abcam, Cambridge, UK; MBL International Corporation, Woburn, MA, USA.

## References

[1] Mizushima N., Komatsu M. (2011). Autophagy: Renovation of cells and tissues. Cell.

[2] Wang F., Muller S. (2015). Manipulating autophagic processes in autoimmune diseases: A special focus on modulating chaperone-mediated autophagy, an emerging therapeutic target. Front. Immunol..

[3] Yang Z., Klionsky D.J. (2010). Eaten alive: A history of macroautophagy. Nat. Cell Biol..

[4] Weidberg H., Shvets E., Elazar Z. (2011). Biogenesis and cargo selectivity of autophagosomes. Annu. Rev. Biochem..

[5] Kaushik S., Cuervo A.M. (2012). Chaperone-mediated autophagy: A unique way to enter the lysosome world. Trends Cell Biol..

[6] Cuervo A.M., Wong E. (2014). Chaperone-mediated autophagy: Roles in disease and aging. Cell Res..

[7] Ciechanover A., Kwon Y.T. (2015). Degradation of misfolded proteins in neurodegenerative diseases: Therapeutic targets and strategies. Exp. Mol. Med..

[8] Ahlberg J., Glaumann H. (1985). Uptake–microautophagy–and degradation of exogenous proteins by isolated rat liver lysosomes. Effects of pH, ATP, and inhibitors of proteolysis. Exp. Mol. Pathol..

[9] Mijaljica D., Prescott M., Devenish R.J. (2011). Microautophagy in mammalian cells: Revisiting a 40-year-old conundrum. Autophagy.

[10] Heath R.J., Xavier R.J. (2009). Autophagy, immunity and human disease. Curr. Opin. Gastroenterol..

[11] Nagata E., Sawa A., Ross C.A., Snyder S.H. (2004). Autophagosome-like vacuole formation in Huntington’s disease lymphoblasts. Neuroreport.

[12] Gros F., Arnold J., Page N., Décossas M., Korganow A.-S., Martin T., Muller S. (2012). Macroautophagy is deregulated in murine and human lupus T lymphocytes. Autophagy.

[13] Gianchecchi E., Delfino D.V., Fierabracci A. (2014). Recent insights on the putative role of autophagy in autoimmune diseases. Autoimmun. Rev..

[14] Macri C., Wang F., Tasset I., Schall N., Page N., Briand J.-P., Cuervo A.M., Muller S. (2015). Modulation of deregulated chaperone-mediated autophagy by a phosphopeptide. Autophagy.

[15] Sasazawa Y., Sato N., Umezawa K., Simizu S. (2015). Conophylline protects cells in cellular models of neurodegenerative diseases by inducing mammalian target of rapamycin (mTOR)-independent autophagy. J. Biol. Chem..

[16] Mintern J.D., Harris J. (2015). Autophagy and immunity. Immunol. Cell Biol..

[17] Zhu L., Wang H., Wu Y., He Z., Qin Y., Shen Q. (2017). The autophagy level is increased in the synovial tissues of patients with active rheumatoid arthritis and is correlated with disease severity. Mediators Inflamm..

[18] Wang F., Li B., Schall N., Wilhelm M., Muller S. (2017). Assessing autophagy in mouse models and patients with systemic autoimmune diseases. Cells.

[19] Muller S., Brun S., René F., de Sèze J., Loeffler J.P., Jeltsch-David H. (2017). Autophagy in neuroinflammatory diseases. Autoimmun. Rev..

[20] Hughes R.A.C., Allen D., Makowska A., Gregson N.A. (2006). Pathogenesis of chronic inflammatory demyelinating polyradiculoneuropathy. J. Peripher. Nerv. Syst..

[21] Vucic S., Kiernan M.C., Cornblath D.R. (2009). Guillain–Barré syndrome: An update. J. Clin. Neurosci..

[22] Zhou S., Chen X., Xue R., Zhou Q., Hu P., Ouyang X., Dai T., Zhu W., Tian S. (2016). Autophagy is involved in the pathogenesis of experimental autoimmune neuritis in rats. Neuroreport.

[23] Alirezaei M., Fox H.S., Flynn C.T., Moore C.S., Hebb A.L.O., Frausto R.F., Bhan V., Kiosses W.B., Whitton J.L., Robertson G.S. (2009). Elevated ATG5 expression in autoimmune demyelination and multiple sclerosis. Autophagy.

[24] Igci M., Baysan M., Yigiter R., Ulasli M., Geyik S., Bayraktar R., Bozgeyik İ., Bozgeyik E., Bayram A., Cakmak E.A. (2016). Gene expression profiles of autophagy-related genes in multiple sclerosis. Gene.

[25] Cai P.-P., Wang H.-X., Zhuang J.-C., Liu Q.-B., Zhao G.-X., Li Z.-X., Wu Z.-Y. (2014). Variants of autophagy-related gene 5 are associated with neuromyelitis optica in the Southern Han Chinese population. Autoimmunity.

[26] Beaino W., Trifilieff E. (2010). Thiopalmitoylated peptides from the peripheral nervous system myelin p0 protein: Synthesis, characterization, and neuritogenic properties. Bioconjugate Chem..

[27] Brun S., Beaino W., Kremer L., Taleb O., Mensah-Nyagan A.G., Lam C.D., Greer J.M., de Seze J., Trifilieff E. (2015). Characterization of a new rat model for chronic inflammatory demyelinating polyneuropathies. J. Neuroimmunol..

[28] Schmalbruch H. (1986). Fiber composition of the rat sciatic nerve. Anat. Rec..

[29] Shehab S.A., Atkinson M.E., Payne J.N. (1986). The origins of the sciatic nerve and changes in neuropeptides after axotomy: A double labelling study using retrograde transport of true blue and vasoactive intestinal polypeptide immunohistochemistry. Brain Res..

[30] Rigaud M., Gemes G., Barabas M.-E., Chernoff D.I., Abram S.E., Stucky C.L., Hogan Q.H. (2008). Species and strain differences in rodent sciatic nerve anatomy: Implications for studies of neuropathic pain. Pain.

[31] Asato F., Butler M., Blomberg H., Gordh T. (2000). Variation in rat sciatic nerve anatomy: Implications for a rat model of neuropathic pain. J. Peripher. Nerv. Syst..

[32] Mizushima N., Yoshimori T., Levine B. (2010). Methods in mammalian autophagy research. Cell.

[33] Tasdemir E., Galluzzi L., Maiuri M.C., Criollo A., Vitale I., Hangen E., Modjtahedi N., Kroemer G. (2008). Methods for assessing autophagy and autophagic cell death. Methods Mol. Biol..

[34] Klionsky D.J., Abdelmohsen K., Abe A., Abedin M.J., Abeliovich H., Arozena A.A., Adachi H., Adams C.M., Adams P.D., Adeli K. (2016). Guidelines for the use and interpretation of assays for monitoring autophagy (3rd edition). Autophagy.

[35] Rubinsztein D.C., Cuervo A.M., Ravikumar B., Sarkar S., Korolchuk V., Kaushik S., Klionsky D.J. (2009). In search of an “autophagomometer”. Autophagy.

[36] Yerra V.G., Kumar A. (2017). Adenosine monophosphate-activated protein kinase abates hyperglycaemia-induced neuronal injury in experimental models of diabetic neuropathy: Effects on mitochondrial biogenesis, autophagy and neuroinflammation. Mol. Neurobiol..

[37] Kosacka J., Nowicki M., Blüher M., Baum P., Stockinger M., Toyka K.V., Klöting I., Stumvoll M., Serke H., Bechmann I. (2013). Increased autophagy in peripheral nerves may protect Wistar Ottawa Karlsburg W rats against neuropathy. Exp. Neurol..

[38] Qu L., Zhang H., Gu B., Dai W., Wu Q., Sun L., Zhao L., Shi Y., Liang X. (2016). Jinmaitong alleviates the diabetic peripheral neuropathy by inducing autophagy. Chin. J. Integr. Med..

[39] Huang H.-C., Chen L., Zhang H.-X., Li S.-F., Liu P., Zhao T.-Y., Li C.-X. (2016). Autophagy promotes peripheral nerve regeneration and motor recovery following sciatic nerve crush injury in rats. J. Mol. Neurosci..

